# Raman Microspectroscopy Analysis in the Treatment of Acanthamoeba Keratitis

**DOI:** 10.1371/journal.pone.0072127

**Published:** 2013-08-20

**Authors:** Giulia Rusciano, Paola Capriglione, Giuseppe Pesce, Salvatore Del Prete, Gilda Cennamo, David Di Cave, Luciano Cerulli, Antonio Sasso

**Affiliations:** 1 Dipartimento di Fisica, Università 'Federico II', Complesso Universitario Monte S. Angelo, Napoli, Italy; 2 Dipartimento di Neuroscienze e Scienze Riproduttive ed Odontostomatologiche, Univ. 'Federico II', Napoli, Italy; 3 Dipartimento di Medicina Sperimentale e Chirurgia, Univ. Tor Vergata, Roma, Italy; 4 CNR, Ist Nazl Ott (INO), Pozzuoli, Italy; University of Leeds, United Kingdom

## Abstract

Acanthamoeba keratitis is a rare but serious corneal disease, often observed in contact lens wearers. Clinical treatment of infected patients frequently involves the use of polyhexamethylene biguanide (PHMB), a polymer used as a disinfectant and antiseptic, which is toxic also for the epithelial cells of the cornea. Prompt and effective diagnostic tools are hence highly desiderable for both starting early therapy and timely suspension of the treatment. In this work we use Raman microspectroscopy to analyse in vitro a single Acanthamoeba cell in cystic phase. In particular, we investigate the effect of PHMB at the single-cell level, providing useful information on both the underlying biochemical mechanism and the time frame for Acanthamoeba eradication in ocular infections. Furthermore, we demonstrate that Raman spectroscopy, in conjunction with standard multivariate analysis methods, allows discriminating between live and dead Acanthamoebas, which is fundamental to optimizing patients’ treatment.

## Introduction

Acanthamoeba keratitis (AK), first recognized in 1973, is a rare, sight threatening parasitic infection, which can result in permanent visual impairment or even blindness [1], [2]. It can occur in patients of any age, sex or race, but mostly manifests in young, healthy adults. In USA 90% of cases of AK is associated with the use of soft contact lens. However, elsewhere in the world, numerous cases of Acanthamoeba (*A.*) have been described in non-contact lens wearers. Moreover, contamination of the contact lens storage case by *A.* are reported in up to 8% of asymptomatic contact lens wearers [3]. At present, there is no consensus therapy for keratitis from *A.*, and the most commonly used topical agents are chlorhexidine and PHMB [4]–[9], which inhibits membrane function. As a matter of facts, being the prognosis in AK directly related to timely diagnosis [10], new methods to ensure a rapid identification of this infection are strongly needed.

Diagnosing AK is a clinical challenge. Initial symptoms resemble those of bacterial or herpetic keratitis, although the clinical management of AK differs substantially. The standard diagnosis in suspect cases is made by culture and isolation of organisms from a corneal culture or detection of trophozoites and/or cysts using histopathology. This requires invasive tests such as corneal scrapping or collection of a corneal biopsy. For laboratory diagnosis, a variety of stains are used, which include haematoxylin and eosin, Gram, Giemsa, methenamine silver or periodic acid-Schiff, and special stains like calcofluor white and acridine orange. These stains are used on corneal scraping, impression cytology and biopsy in conjunction with culture to facilitate the diagnosis. The association of a microscopic examination of the corneal scraping and a culture procedure is the most commonly used diagnostic procedure. This strategy is far from being adapted to the urgency of the situation because the complete procedure requires 21 days of culture and presents a very low overall sensitivity (50% misdiagnosed cases). Thus, a negative culture does not necessarily rule out *A.* infections. Molecular biology and more precisely PCR procedures amplifying *A.* DNA have been also developed to improve AK management. Molecular diagnostic assays have proved to be more rapid, as well as more sensitive and specific than smear and culture, but they still require corneal scraping to obtain the samples, together with trained personnel. On the other hand, PCR techniques are expensive, requiring skilled technicians and expensive kit, and this limits wide usage worldwide. More importantly, PCR does not recognize living parasites from dead forms so it does not give to clinicians sufficient information for a timely suspension of the *medical treatments*, which is also toxic to the epithelial cells of the cornea.

In this frame, Raman Spectroscopy (RS) can play an innovative role. This technique has emerged in recent years as a powerful techniques for non-invasive characterization of a wide variety of biological materials, ranging from tissues to single cell or even single bio-molecules. It has been widely employed in an impressive number of studies of both in vitro and in vivo biological systems (DNA, peptides and proteins, microbes and viruses, cancer cells, etc.) [11]–[18]. Medical applications of RS are also very numerous and have been described in review papers [19], [20] and textbooks [21]. In particular, Erckens *et al.* [22] had given a review of RS applied to ophthalmology.

In this work we discuss of a new application of RS in ophtalmology, related to some important aspects of AK medical treatment. First of all, we investigate the effect of PHMB at single cell level, providing useful information on both the underlying biochemical mechanism and the time frame for *A.* eradication in ocular infections. Furthermore, we demonstrate that RS allows discrimination between live and dead cells, which is fundamental for a timely suspension of AK patients treatment with PHMB. This second part of our study is based on the use of Principal Component Analysis (PCA) [23], a powerful statistical analysis, which enable highlighting subtle differences in the Raman spectra of similar samples.

## Materials and Methods

### Raman Confocal Microscopy

Raman spectra were acquired with a Raman confocal microscope (Witec, *alpha 300*). The system is equipped with an excitation source at 532 nm, provided by a frequency doubled Nd:YAG laser. For the measurements, a 60X objective was employed, providing a diffraction-limited spot on the sample. Raman scattered light was collected with the same objective lens and guided to the spectrograph by a 50-*µ*m core optical fibre, which assures system confocality. All the spectra were acquired over the spectral range from 700 to 1800 cm^−1^, with a spectral resolution of ∼1 cm^−1^, as estimated by the FWHM of the 1001 cm^−1^ polystyrene band. To avoid sample photodamage, the power impinging on the sample was limited to 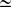
200 *µ*W. For single spectra acquisition, the Raman signal was integrated for 10 s.

### Principal Components Analysis

PCA is a widely used statistical tool for the analysis of large spectral data sets. It allows the reduction of the number of variables of a multidimensional dataset, retaining, at the same time, most of the variation within the data. As a matter of facts, PCA decomposes a data set into "principal components" (PCs), so that the relevant information originally contained in a large amount of variables is condensed in a very small number of PCs (typically less than 10) [23]. In particular, when applied to Raman spectra, PCA condenses the information contained in each spectrum (which has a number of observables equal to its points) in only a few (3 in our case) variables (PCs scores), so that each spectrum can be represented as a point in a 3-dimensional space (scores plot). The procedure of data transformation involves the diagonalization of the correlation matrix of the initial data; as a result, the new observables (PCs) are uncorrelated data, and carrie the most relevant information to differentiate among the initial Raman spectra. That makes particularly advantageous to highlight even subtle differences between spectral features which are masked by noise. To establish the correct number of PCs to be chosen, we calculated the percent of the total variability explained by each PC. From these data, we verified that the cumulative sum of the first three PCs explains more than 97% of the total variability, so that it is reasonable to retain only these 3 components to reduce the dimensions of the initial data. The differentiation efficiency of PCA can be evaluated by observing the distribution of PC scores in the scores plot. Ideally, Raman spectra of different samples should occupy well-separated regions of the scores-plot. In this work, PCA was performed on Raman spectra using an home-made MATLAB routine, based on the use of the *princomp* Mathlab routine. Before analysis, Raman spectra were background-corrected by removing a forth-order polynomial and eliminating spurious signals deriving from cosmic rays contributions.

### Cell Culture Protocol

*A.s* used in this experiment were isolated from the surface of a contact lens previously employed by a AK patient. Since *A.s* were not directly isolated from humans, we deemed unnecessary any approval from the ethics committee. Protozoa were suspended in Page€s amoeba saline and centrifuged at 800×g for 5 minutes according to ref. [1]. The sediment was suspended and isolation of Acanthamoeba was performed on 1,5% non nutrient agar (Difco, Milan, Italy) enriched by Escherichia coli K12. Plates were stored at 4°C for molecular studies. Isolate DNA extractions from non-nutrient agar plates were performed using QIAamp DNA Micro Kit (Qiagen, Italy) and stored at 20°C. A 405-bp region of the 18S-rRNA gene (ASA.S1) that includes the Diagnostic Fragment 3 (DF3) was amplified using the genus-specific primers JDP1 and JDP2 [24] to a final volume of 25 *µ*l. The PCR program was as follows: denaturing step at 96°C for 2 min, followed by 35 cycles of denaturing for 1 min at 96°C, annealing for 1 min at 60°C and extension for 1 min at 72°C, followed by a final extension at 72°C for 7 min. Sterile distilled water was included as negative control in each batch of DNA extraction and PCR reactions. Bands were visualised on SYBR Safe*^TM^* DNA gel stain (Invitrogen Corporation, Italy) stained 1% agarose gels. PCR products were purified using Nucleo Spin Extract II (Macherey-Nagel GmbH & Co. KG ) purification kit and sequenced for both strands by the Bio-Fab Research (Italy). Assignment to genotypes was performed by sequence comparison and phenetic analysis. All the sequences revealed a strict correspondence with the T4 genotype.

## Results and Discussion

### *A.* Raman Spectrum

**Figure 1:**
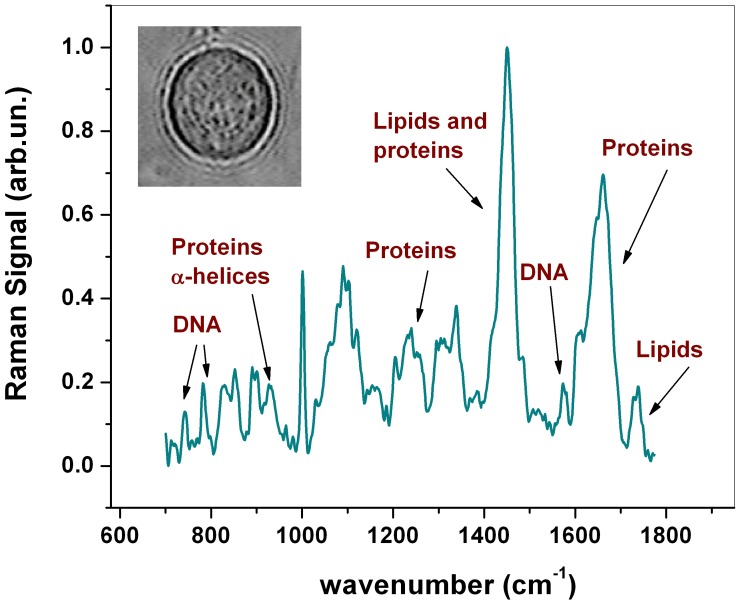
Raman spectrum from a live *A*. Illustration of an average Raman spectrum in the 700–1800 cm^−1^ region from a live Acanthamoeba (cystic phase) obtained from 10 acquisitions at a randomly selected location inside the cell. Significant peaks and their relative assignments are labeled. The inset shows an optical image of the analysed *A*.

Figure 1 shows a typical Raman spectrum obtained in this work in the fingerprint region from 700 to 1800 cm^−1^. It was obtained by averaging 10 spectra measured at a randomly selected location inside the cell (cystic phase). The cell was kept in a standard PBS buffer solution. A tentative assignment of the prominent peaks is presented in Table 1 [25]–[26]. The strongest feature at around 1440 cm^−1^ corresponds mainly to C-H vibrations in both lipids and proteins. The relatively broad feature around 1640 and 1260 cm^−1^ are due to proteins and correspond to Amide I and Amide III band (respectively), which have been shown to be sensitive to the secondary structure of the proteins. Information on protein secondary structure can be inferred also from the sharp peak around 936 cm^−1^, due to *α*-helix C-C backbone stretching. Some more peaks corresponding mainly to amino acids can be found around 1001 cm^−1^ (phenylalanine, Phe) and 854 cm^−1^ (tyrosine, Tyr). Peaks due mainly to lipids are present at 1301 cm^−1^ and 1735 cm^−1^, the latter corresponding to C = O ester. Finally, several peaks in the spectra can be attributed to nucleic acids, corresponding to both single nucleotides (783 cm^−1^ and 1578 cm^−1^) and sugar-phosphate backbone vibrations (828 cm^−1^).

**Table 1:** Observed Raman frequencies with corresponding band assignments for *A*.

Wavenumber (cm^−1^)	Component	Tentative Assignment
783	DNA	U,C,T ring
788	DNA	O-P-O sym. str.
854	Proteins	Ring br. Tyr
936	Proteins	C-C str. *α*-helix
1001	Proteins	Sym. Ring br Phe
1060–1100	DNA/Lipids	PO 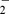 str./C-C str.
1210–1290	Proteins	Amide III
1301	Lipids	CH_2_ twist
1420–1480	DNA/Proteins/Lipids	CH def
1578	DNA	G, A
1620–1680	Proteins	Amide I
1736	Lipids	C = O ester

Assignment is mainly based on refs. [27] and [31].

### Monitoring the Effect of PHMB at Single Cell Level

As a first step, we tested the ability of Raman micro-spectroscopy to monitor the effect of polyhexamethylene biguanide (PHMB) eye drop (0.02% concentration) on single *A.* PHMB is the first antiseptic known to have a specific mechanism of action [9]. PHMB acts by binding of its highly charged positive molecules to the mucopolysaccharide plug of the ostiole. This results in penetration through the ostiole to the internalized amoeba, where the drug binds to the phospholipids bilayer of the amoeba cell membrane causing membrane damage, cell lysis, and death. The lethal action of this compound is, therefore, due primarily to the irreversible loss of essential cellular components through the damaged plasmalemma of the amoeba.

We have considered two different *in vitro* experiments. In a first simpler approach (*case a*), *A.*s were permanently placed in PHMB solution, while in a second approach (*case b*) *A.*s were intermittently exposed to PHMB.

The results of these measurements are reported below.

#### Case a

**Figure 2:**
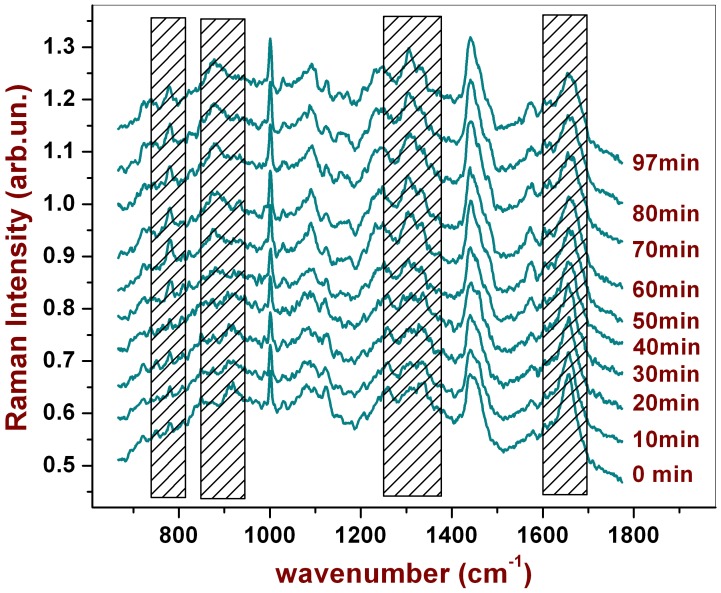
Raman spectra from an *A*. acquired in the same point (cytoplasmatic region) after different time intervals of continuous cell exposition at PHMB. The dashed spectral regions refer to peaks which exhibit significant changing during PHMB exposure.

In this case, the Raman probe was focused into the cell cytoplasm and spectra were acquired every 2.5 minutes in the same point. The integration time for each spectrum was 10 s. and the power impinging on the sample was 200 *µ*W. To rule out any possible photo-induced effect, the same set of measurements was repeated for a cell not exposed to PHMB (control measurement). Figure 2 shows the spectra acquired at selected times. Cosmic rays were removed and a Savitsky Golay filter (averaging on 5 points) was applied to smooth the spectra for a better visualization.

**Figure 3:**
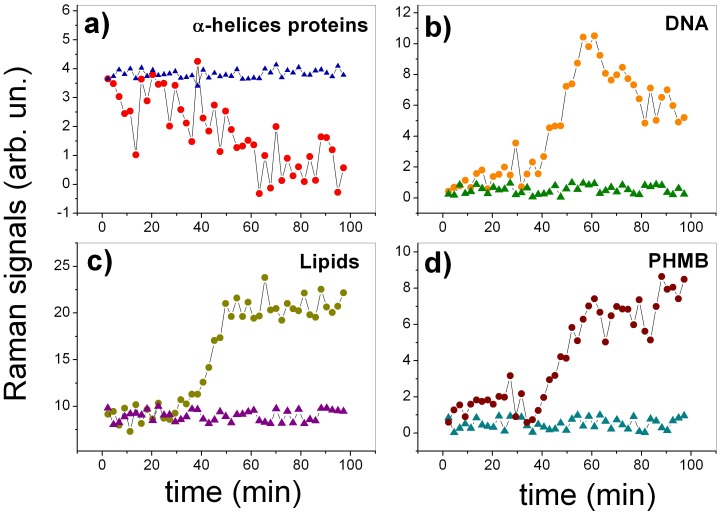
Time evolution of selected Raman peaks for a cell exposed to PHMB (dots) and for the control cell (triangles). Panels a), b), c) and d) report the behavior of pecks at 936 cm^−1^ (*α*-helix proteins), 788 cm^−1^ (DNA sugar backbone), 1301 (Lipids) and 877 (PHMB), respectively.

**Figure 4:**
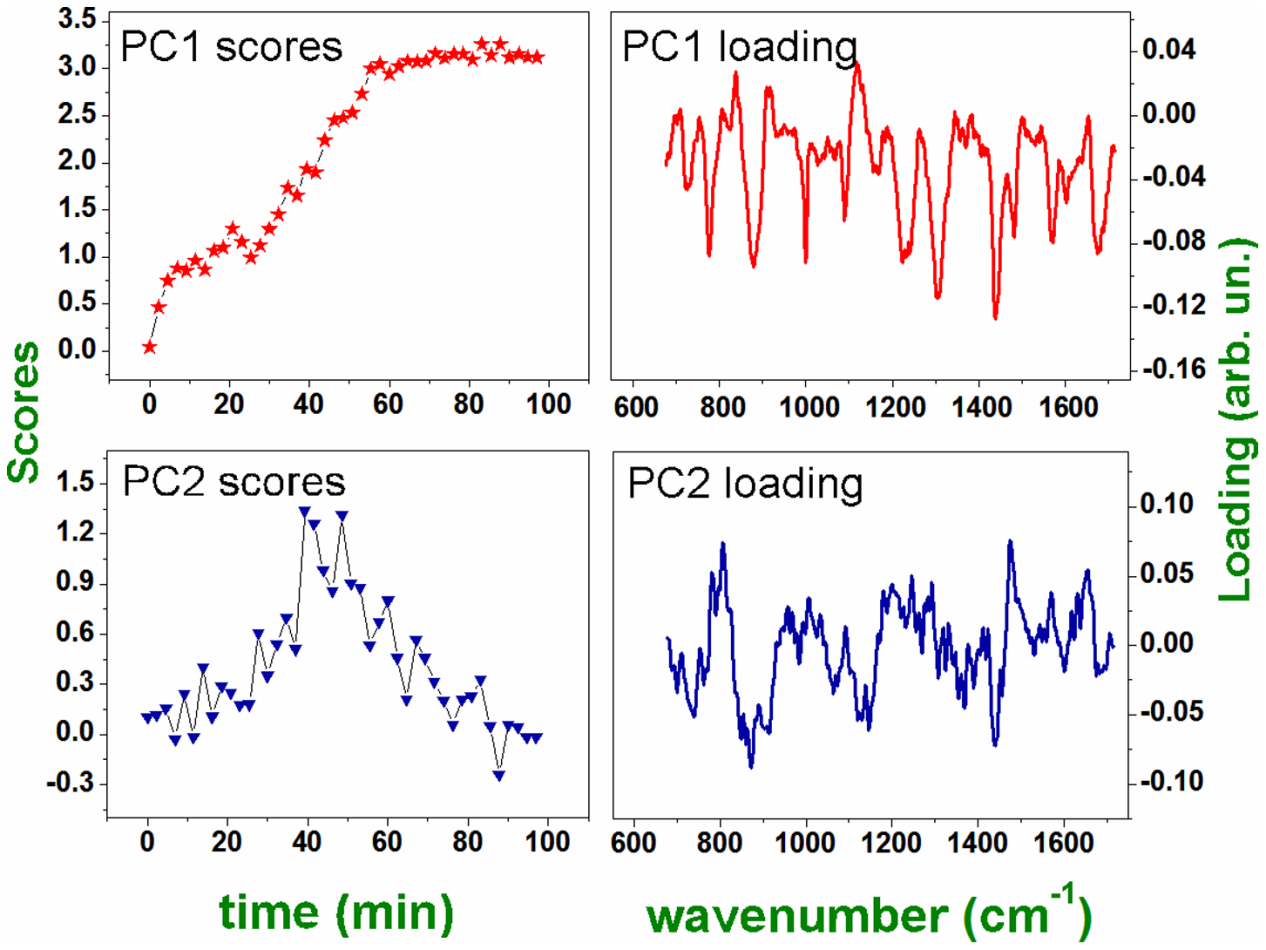
PCA analysis for the spectra acquired in the same point after different times (see text for details). a) PC1 and PC2 scores it vs time for the time series of Raman spectra of a single cell exposed to PHMB. b) PC1 and PC2 loading for the same spectra.

As it is possible to see, the intensity of several peaks changes as the time passes by; the most evident variations are highlighted in Fig. 2 by the shaded region. A quantitative evaluation of this effect has been obtained by subtracting from each spectrum a forth order polynomial baseline and, therefore, measuring the intensity of the peaks that appeared to change. Figure 3 reports the results of this analysis for the peak at 936 cm^−1^ (*α*-helix proteins), 788 cm^−1^ (DNA sugar backbone), 1301 (Lipids) and 877 (PHMB): dots correspond to measurements performed on the cell exposed to PHMB, while triangles correspond to the control. Interestingly, while the peaks of the control cell remain constant throughout all the analyzed time period, many Raman peaks show a significant change when the cell is exposed to PHMB. In particular, it is possible to observe a significant change for many peaks after 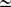
30 minutes, suggesting evidently a correlation of the observed events. All these changes seem to reflect the complex biochemical evolution inside the cell occurring during cell death. Notably, there is a constant decreasing of the peak corresponding to *α*-helix of more than 80% (3.a), suggesting an event of protein degradation or protein denaturation [27], [28]. Contemporaneously, there is an increasing of the lipid band at 1301 cm^−1^ (3.c), probably due to the formation of lipid vesicles inside the cytoplasm, due to the strongly hostile environment in which the cell lies. Similar behaviors were observed by Verrier *et al.* [29] in human lung carcinoma epithelial cell (A549) when cell death was induced by Triton X-100, a toxic agent. Moreover, our data show the arising of a peak at 877 cm^−1^ in the spectrum (3.d), which corresponds to PHMB solution, as verified by acquiring the Raman signal from the pure sample (data not shown). This behavior suggests that PHMB penetrate through the cell membrane, reaching the confocal region where the Raman signal is acquired. Although the PHMB infiltration starts immediately, it seems to be accelerated after ∼40 minutes, probably due to a cytoplasmic membrane breakdown. Our data also suggest significant changes in the cell nucleus. The most significant change, involves the phosphodiester bond vibration at 788 cm^−1^ (3.b). In particular, the measured spectra indicate a strong increasing of this peak (initially not observed in the spectra) after 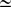
30 minutes, followed by a reduction of the peak intensity of more than 40% with respect to the maximum observed value. Globally, this behavior could be explained in terms of the overlapping of two events: the first involves the breakdown of the nuclear membrane, which gives rise to nuclear fragmentation and DNA diffusion into the cytoplasm and, consequently, the appearance of the peak at 788 cm^−1^ in the spectrum. However, cell death involves the breaks of phosphodiester bonds and DNA disintegration, which explains the decreasing of the peak observed after 40 minutes. To check this hypothesis, we have tried to deconvolute the two phenomena by analyzing the Raman spectra time series by PCA. The results of this analysis are reported in Fig. 4. Interestingly, PCA recognizes the separate contribution of two co-factors: the first involves the evolution of peaks of PC1 loading and increases with time (see PC1 scores *vs* time), the second factor, representing the evolution of peaks of PC2 loading (uncorrelated from the variation of peaks described by PC1) has a wave-like behavior. Therefore, it is reasonable to assume that while PC1 describes the biochemical modification induced by PHMB, PC2 describes the effect of the wave of cellular debris reaching the confocal detection volume. Coherently with this assumption, the peak at 788 cm^−1^ is negative in the PC1 loading (describing, therefore, the reduction of the peak associated with DNA backbone disruption), while it is positive in the PC2 loading (representing the incoming of DNA into the Raman probe spot).

#### Case b

In this second case, the cell under investigation was placed in an open chamber and intermittently exposed to PHMB. The motivation of this analysis arises from the necessity to reproduce, as close as possible, the conditions occurring *in vivo* in ophthalmic patients affected by AK. In fact, due to the tear flux (∼1–2 *µ*l/min), the total volume of tear film (7–9 *µ*l in a normal individual) is constantly refreshed. On average, therefore, it is reasonable to assume that, when a PHMB solution is applied on eye, it remains in contact with *A.* present on the cornea surface (or just below) for not more than five minutes before the complete washing of cell environment. Medical prescriptions for patients affected by Acantamoeba keratitis involve the application of one drop (∼5 *µ*l) every hour (in the attack phase), so that we analyzed the response of cells intermittently exposed to a 1∶2 concentration of PHMB solution in PBS.

**Figure 5:**
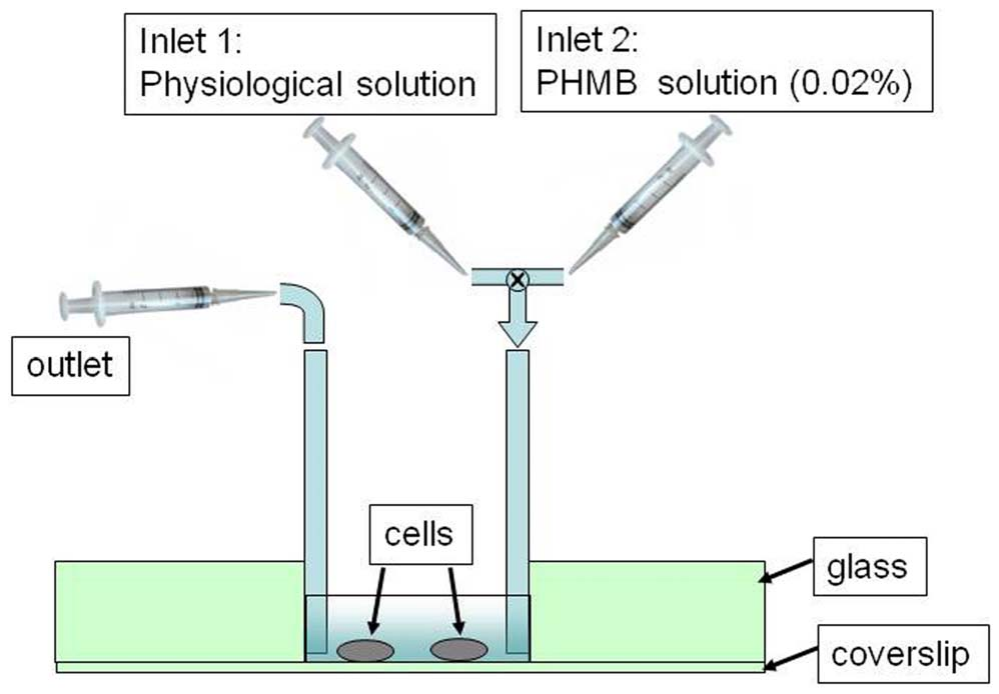
Sketch of the cell chamber used to reproduce the therapy of PHMB in an infected eye (see text to explanation).

**Figure 6:**
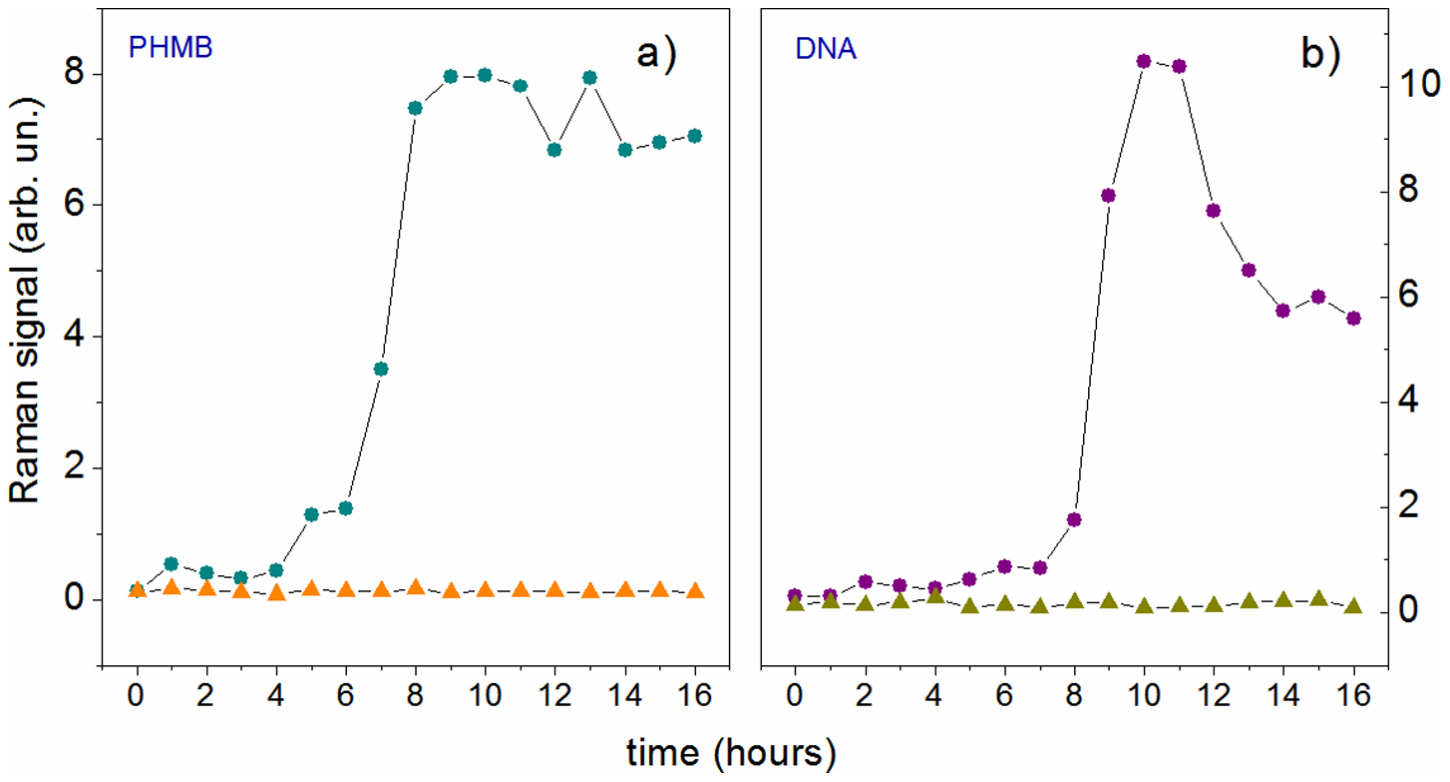
Time evolution of PHMB (panel a) and DNA (panel b) signals detected in the confocal region of the Raman probe. Dots and triangles refer to a cell *intermittently* exposed to PHMB (dots) and the control cell (triangles), respectively.

Figure 5 reproduces a sketch of the chamber used in the experiment. Two soft rubber tubes were glued to the wall chamber and used as inlet and outlet fluid ways. At the beginning, we introduced in the cell chamber only the PBS solution containing *A.*s and acquired the Raman spectrum from the selected cell (reference spectrum). Therefore, with a syringe, we gently extracted the PBS buffer solution from the chamber and, with a second syringe, we introduced the PHMB solution. This operation was done taking care that the *A.* selected for the Raman investigation remained in the same position to guarantee that also in this scheme Raman spectra were acquired in the same cell position. *A.* was exposed to PHMB for only five minutes (which corresponds to the period requested to refresh completely the content of tear fluid in a healthy eye), after which it was washed by gentle aspiration of the liquid in the chamber and successive reintroduction of fresh PBS buffer into the chamber. This treatment was repeated every hour for 16 hours. After every washing treatment a Raman spectrum was acquired. Figure 6 reports the results of this analysis. The experimental outcomes found with this second procedure essentially confirm those already discussed in the previous case. Interestingly, all the Raman peaks exhibit similar behaviors, as in Fig. 4. For sake of simplicity, in Fig. 6 we just report the temporal behavior of PHMB and DNA peaks inside the confocal region. However, in this case, the time scale seems to be ruled by the it effective time of *A.* exposure to PHMB, with only minimal (if any) effect of cell recovery when PHMB is washed away. For instance, Fig. 6 shows that when PHMB is intermittently administered, membrane rupture occurs only after ∼6 hours, which correspond to ∼30 minutes of effective exposure to PHMB. Consistently, also DNA appearance in the confocal region is delayed to ∼8 hours.

### Live versus Dead Cells

**Figure 7:**
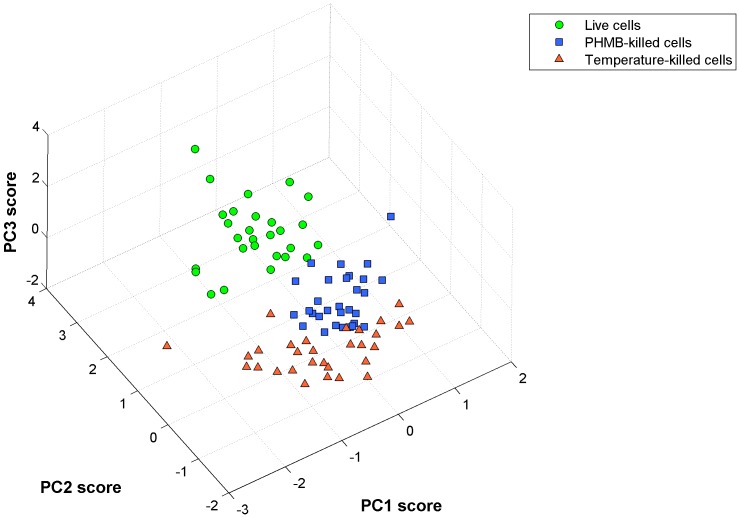
PCA analysis of live and dead protozoa (cystic phase). Dots, squares and triangles correspond to o live, PHMB- killed and temperature-killed cells, respectively.

As discussed above, one of the main issue that remains to be addressed in the therapeutic management of ophthalmic patients affected by AK is discrimination between live and dead cells obtained from eye-microsurgery. To establish if Raman spectroscopy is able to asses this topic, we analyzed three classes of cells: *i)* live *A.* in PBS buffer, *ii)* cells exposed for 24 hours at PHMB (0.02%, 1∶2 concentration), and *iii)* cells exposed to water at 80° for 5 minutes. Viability tests, performed for control, revealed that the latter two classes correspond to dead cells. So far, single cell spectra were acquired from 30 cells for each class. During acquisition, the Raman probe, at a power of 100 *µ*W, was focused on the cell nucleus. Spectra were collected in the 800–1700 cm^−1^ region, with an integration time of 20 s. Spectra were therefore analysed by PCA. Figure 7 shows the score plot relative to this analysis: dots correspond to live cell, while squares and triangles correspond to PHMB- and temperature-killed cells. As it is possible to see, there is a marked capability of Raman spectroscopy to separate live and dead cells, while there is some overlapping between the two classes of killed cells. The predictive capability of Raman spectroscopy was quantitatively evaluated by applying the leave-one-out cross-validation (LOOCV) procedure [30]. This analysis revealed that dead cell can be discriminated with respect to live cells with both high sensitivity and high specificity (both around 97%), suggesting the effectiveness of Raman analysis as a diagnostic tool in ophthalmology, even for clinical applications.

## Conclusions

The results of this study demonstrate that RS has great potential to become an effective tool for the diagnosis of AK and for establishing the effectiveness of its clinical treatment. Specifically, we analysed the effect of PHMB eye drops at single cell level. By observing the variation of selected Raman bands due to DNA, lipids and proteins in *α*-helices configuration, we reconstructed the cellular biochemical path induced by PHMB. Our results suggest that the action of this antiseptic starts from the rupture of the cytoplasmatic membrane of the protozoa, after which this leads to protein and DNA degradation and, therefore, to cell death. This study has been performed by simulating, as close as possible, the conditions occurring in vivo in an ophthalmic patient affected by AK, for which PHMB administration is diluted by the tear flux. Furthermore, we have demonstrated that the *fingerprint* character of *A.* Raman spectrum, which uniquely identifies this cellular specie, allows also discrimination between live and dead protozoa, with both high sensitivity and specificity (97%). This feature represents a significant improvement with respect to molecular diagnostic assays (such as PCR) commonly used for AK diagnosis and treatment monitoring.

The investigation presented in this paper could be easily extended to other infective organisms, which could pave the way for the use of RS as a rapid, cheap and effective diagnostic tool in ophthalmology, even for clinical applications.
